# Does NETosis Contribute to the Bacterial Pathoadaptation in Cystic Fibrosis?

**DOI:** 10.3389/fimmu.2014.00378

**Published:** 2014-08-11

**Authors:** Samir Rahman, Mihaela Gadjeva

**Affiliations:** ^1^Division of Infectious Diseases, Department of Medicine, Brigham and Women’s Hospital, Harvard Medical School, Boston, MA, USA

**Keywords:** neutrophils, NETs, histones, neutrophil elastase, chromatin, cystic fibrosis, *Pseudomonas aeruginosa*

## Abstract

Significant advances in our understanding of neutrophil biology were made in the past several years. The exciting discovery that neutrophils deploy neutrophil extracellular traps (NETs) to catch pathogens paved the way for a series of additional studies to define the molecular mechanisms of NET generation and the biological significance of NETosis in acute and chronic pathologic conditions. This review highlights the latest knowledge regarding NET structures, deployment, and function, with an emphasis on current understanding of NET proteomes, their conservation, and significance in the context of cystic fibrosis (CF), a condition characterized by excessive extracellular DNA/NET presence. We also discuss how our understanding of NETosis yields novel therapeutic approaches and their applicability to CF.

## Introduction

The significance of the role of neutrophils in infection response has been appreciated since the latter part of the nineteenth century. For decades, it was understood that neutrophils primarily act in one of two different ways upon activation: by releasing antimicrobial proteins into extracellular space through degranulation, or through phagocytosis of invading microbes. However, a recent focus, spurred by the identification of neutrophil extracellular traps (NETs), demonstrated yet another significant role for neutrophils (1). NETs represent an innate immune response to bacteria, fungi, and viruses (2). They have also been implicated in various pathologies and are a significant area of study with the potential to create novel therapies to a variety of immunological diseases.

## NET Core Proteome

Extracellular DNA serves as the physical scaffold upon which the NET-associated proteins are attached to facilitate adhesion and sequestration of pathogens, such as bacteria (3). The components that comprise the highest proportion of NET protein content are histones. Specifically, histones H2A, H2B, and H3 are present in the high amounts of 379.3, 298.9, and 199.2 mg per gram of NET DNA, which represents molar percentages of 26.29, 23.95, and 14.50%, respectively (2). The functional significance of a substantial histone presence is that it represents a potential attachment site for pathogens and carries antibacterial activity (4). The remainder of proteins that decorates the NET DNA scaffold include granular proteins, cytoplasmic proteins, cytoskeletal proteins, and metabolic enzymes. They are present in far smaller proportions. For example, myeloperoxidase (MPO) is present at 71.3 mg per gram of NET DNA, or a 1.01% molar amount (2). Granular proteins include antimicrobial agents such as lactotransferrin and cathepsin G. Metabolic enzymes include alpha-enolase and transketolase (2).

Neutrophil extracellular traps are released in response to various stimuli such as rheumatoid factor, IgG, TNF, or bacterial pathogens, such as *Pseudomonas aeruginosa* (6, 5, 7). Although variations within the protein compositions of the NET proteomes were reported, significant commonality was present in the repertoire of the NETs deployed in response to various triggers. Nineteen constituent proteins were consistently found in NET structures, regardless of stimulus. These conserved proteins included β-actin, α-actinin-1/4, α-enolase, filamin-A, histone H2A, histone H2B, histone H4, lactoferrin, MPO, myosin-9, moesin, neutrophil defensin 2, neutrophil elastase, neutrophil gelatinase-associated lipocalin, plastin-2, profilin-1, resistin, glucoso-6-phosphate isomerase, and transketolase (Table 1) (6). Because of this conservation of the repertoire, we propose to term this NET-associated assembly the NET “core proteome”.

**Table 1:** **NET-reported proteomes**.

Cellular Compartment	NET-Constituent Protein	PMA	PA01	TNF	RA	IgG
Granules	*Leukocyte elastase*	+	+	+	+	+
	*Lactotrensferrin*	+	+	+	+	+
	*Azurocidin*	+	+	+	−	+
	*Cathepsin G*	+	+	+	−	+
	MPO	+	+	+	+	+
	Leukocyte Proteinase	+	−	−	−	+
	*Lysozyme*	+	+	+	+	−
	*Neutrophil Defensins*	+	+	−	+	+
	*Resistin*	−	−	+	+	+
Nucleus	*Histone H3*	+	+	−	−	+
	*Histone H2B*	+	+	+	+	+
	*Histone H2A*	−	+	+	+	+
	*Histone H4*	+	+	+	−	+
Enzymes	α*-enolase*	+	+	+	+	+
	*Transketolase*	+	+	+	+	+
	*GAPDH*	−	+	+	+	+
	*GPI*	−	−	+	+	+
	Aldolase	−	+	−	−	−
Cytoplasm	*Protein S100A9*	+	−	+	+	+
	*Protein S100A8*	+	−	+	+	+
	*Protein S100P*	−	−	+	+	+
	*Plastin-2*	−	−	+	+	+
	*Filamin-A*	−	−	+	+	+
Peroxisome	*Catalase*	+	+	−	+	−
Cytoskeleton	*Actin (β/γ)*	+	+	+	+	+
	*Myosin-9*	+	+	+	+	+
	α*-actinin (1/4)*	+	+	+	+	+
	*Profilin*	−	−	+	+	+
	*Moesin*	−	+	+	+	+
Membrane	*Neutrophil gelatinase-associated lipocalin*	−	−	+	+	+
	*Shared by three or more stimuli*	18/19	19/20	25/25	23/24	26/27

*Constituent NET proteins are indicated according to cellular localization. Proteins shared by three or more stimuli are italicized*.

With regard to NET proteomic variations, it was reported that rheumatoid factor elicited only six unique proteins, as did IgG, and TNF yielded only three unique proteins (Table 1) (6). Across the distinct stimuli, only 10–20% of decorative proteins were distinctive. The fact that proteomic variations existed was interpreted as an indication that the various stimuli triggered different signaling cascades that yielded the slightly varied results in proteomic outputs. While this is certainly possible, another scenario is that the additional decorative proteins adhered to NETs subsequently. Given the highly adhesive nature of NETs, the structures likely bound slightly different arrays of proteins in the different environments surrounding each stimulus. This view is consistent with the role of NETosis as a component of the innate immune response, which serves as a rapid defense that is not specifically tailored to different pathogens. It is likely that differences in the protein environment that surrounds NETs may lead to the differences in the protein cargo that decorate them. Because of the significant overlap in protein presence on the chromatin structures, we favor the concept that the NETs, when initially cast, carry a conserved core proteome that is not dependent on the stimulus. Once in the extracellular environment, NETs bind additional cargo.

The NET proteome provides insights into NET functionality, in that constituent proteins can be categorized into functional groups. For example, histones H2A, H2B, and H4, given the adhesive nature of histones, serve to physically sequester pathogens such as bacteria. Upon sequestration, microbial killing can be executed through antimicrobial proteins, such as MPO, which generate cytotoxic reactive oxygen species (ROS) (HOCl), or through nutritional immunity involving pathogen starvation of divalent cations mediated by S100 proteins (2, 8). S100A8 and S100A9 starve *Candida albicans* via creating local environments with low divalent ion concentrations, as divalent ion presence is required for *Candida albicans* growth.

The aspect that needs further characterization is the clearance mechanism that follows NETosis. It has been shown that serum DNase I can clear NETs (9). However, whether serum DNase I is systemically or locally upregulated with release of NETs, or whether resident macrophages release DNase I to degrade NETs remains to be explored. An enhanced understanding of the *in vivo* clearance mechanism of NETs would be beneficial in the context of pathologies that involve aberrant or excessive presence of extracellular DNA or NETs. Of note, impaired NET homeostasis was reported in SLE patients and is linked to decreased NET degradation likely due to deposition of anti-DNA antibodies onto NETs, thereby shielding them from DNase I (10).

Interestingly, DNase I-mediated digest of NETs releases active elastase (11). We speculate that this may contribute to the two-step mechanism of NET bactericidal potential, which initially, upon deployment, NETs capture, starves the pathogens, and, secondly, upon partial degradation, releases a battery of antimicrobial peptides to completely inactivate the target. This multi-step killing mechanism is driven by the release of elastase, which preferentially targets histones, enolase, lactoferrin, etc. to generate antimicrobial peptides. Indeed, NETs are equipped with peptidyl prolyl cis-trans isomerase, which is needed for the generation of active histone-derived antimicrobial peptides.

## Missing Links in Our Understanding of How NETosis Occurs

Today, unlike 5 years ago, there is abundant information on stimuli that induce NETosis. These include various pathogenic stimuli and inflammatory mediators (e.g., TNF, IgG, TLR ligands, and complement). For example, activation of Fc-receptors and associated ITAMs can trigger NETosis (12).

However, we know relatively little about the intracellular events resulting in the extracellular expulsion of chromatin. What we do know is that, while not exclusively, to a large extent, NETosis is a ROS-triggered event, dependent on the activity of NADPH oxidase. This is true for a variety of pathogenic stimuli, including LPS, TNF, and bacterial stimulation (e.g., *P. aeruginosa*) that lead to the production of ROS through the enzymatic activity of NADPH oxidase (7, 13).

A downstream event of NADPH oxidative activity is thought to be peptidyl dearginase 4 (PAD4) activation (14). While the molecular details of the pathway remain unclear, it seems that the release of ROS is translated into translocation of PAD4 to the nucleus to modify histones H3 and H4 at Arg 17 and 3, respectively, to convert them to citrulines and hence promote chromatin decondensation (15); a latter step is needed for NET release. This also results in a loss of the characteristically multilobular morphology of the neutrophil nucleus, as demonstrated by electron microscopy of neutrophils upon PAD4 activation (16). To work, PAD4 requires high levels of calcium (17). This requirement of PAD4 is difficult to reconcile with the concentrations of calcium in the cell. So, how does the PAD4 work? It is possible that PAD4 forms complexes with yet unidentified protein partners that lower the requirement of PAD4 for calcium. This scenario is based on the example that PAD4, complexed with antibody, displays lower need for calcium (18). Another possibility is that increased calcium levels result from activation of ITAMs, and such elevated levels of calcium promote the activation of PAD4 (19). Alternatively, compromises in plasma membrane integrity might facilitate extracellular calcium influx, thereby promoting PAD4 activation, consistent with the original model that NETosis is a form of cell death, given that a certain threshold of activation is met. Yet, this concept has been challenged by the observation that after NET release, cells retain mobility (20, 21).

Neutrophils rely heavily on glycolysis and the pentose phosphate cycle for generating ATP and NADPH. The presence of metabolic enzymes on NETs suggests a possible link between metabolic state and NETosis. It is likely that decrease in the influx of glucose slows down glycolysis and triggers compensatory responses such as autophagy activation. Expectedly, there is a link between cellular autophagy and NETosis. Mammalian target of rapamycin (mTOR) regulates NET formation by post-transcriptional control of hypoxia-inducible factor 1α (HIF-1α) in response to LPS (22). Autophagy leads to chromatin decondensation, which precedes NET release (23). Furthermore, a separate study revealed that inhibitors of PAD4 produced a reduction in autophagy activities in an osteocarcinoma cell line (24). Clearly, a positive correlation between the two processes exists.

Infection may lead to nutrient depravation, which is an inducing factor of autophagy (25) Alterations in concentrations of substrate, such as the nutrient glucose, for glycolysis, or the pentose phosphate cycle, is paralleled by alterations in secondary modifications of metabolic enzymes and their locations within the cell. This model comes from the activation mechanisms of the glycolytic enzyme glyceraldehyde-3-phosphate dehydrogenase (GAPDH). GAPDH is of particular interest, as it has been shown to be consistently present on NETs (Table 1) (6). It has been recently elucidated that, upon stress conditions on the cell, GAPDH is translocated from the cytoplasm to the nucleus (26). This translocation occurs as a result of post-translational modifications on the GAPDH molecule. For example, activation of Fc-receptors can trigger ROS production (27). This ROS presence, in turn, yields modifications, through metabolic changes, of GAPDH, including acetylation and nitrosylation, which facilitate its transfer to the nucleus. This model is a reflection of GAPDH forming complexes with nuclear chromatin that is subsequently expelled from the nucleus and the cell upon histone citrullination by PAD4. The question that is raised by the GAPDH model of metabolism-linked translocation to the nucleus is whether PAD4 translocation to the nucleus may follow the same model and be influenced by metabolic state. This notion will need to be studied in order to determine if an intimate link between metabolic stress and PAD4 activation and subsequent NETosis exists.

## Does Our Current Knowledge of NETosis Suggest New Treatment Options for Cystic Fibrosis?

Elevated presence of extracellular DNA is a hallmark of cystic fibrosis (CF), and it is natural to question what proportion, if any, of the extracellular DNA is extracellular trap (ET)-derived. Traditionally, extracellular DNA has been viewed as originating from cellular debris. Indeed, administration of rhDNase (e.g., pulmozyme) does lead to a reduction in the concentration and length of extracellular DNA and is used in clinic for treatment of CF (28). Clues that the extracellular DNA in CF might be ET-derived are emerging from recent studies. Dubios and colleagues produced beautiful images of the characteristic morphological hallmarks of NETosis in neutrophils (loss of lobular nucleus, breakage of nuclear envelope, and decondensation of chromatin) in a patient with CF; unfortunately, quantification of the data was missing (29). Another study, conducted by Papayannopoulos and colleagues, utilized native-PAGE analysis to characterize the chromatin structures in CF sputum samples from 10 CF patients who were not on DNase I therapy. Authors reported that complexes between extracellular DNA, neutrophil elastase, and MPO existed in CF sputum, and that solubilization of sputum depended on NET-released elastase that proteolytically targeted NET-associated histones (11). Authors suggested that as much as 50% of the extracellular DNA in the CF sputum might be NETs; however, while this data was quite exciting, the study provided no patient information. Using LC-MS/MS analysis of NET-enriched samples derived from a cohort of CF patients we showed a significant conservation of NET-associated proteins, again suggesting that NETs were present in CF (5). Clearly, further research is warranted to quantify the ET structures in CF and correlate their presence with disease progression. The existence of conserved NET-associated protein cargo [e.g., NET-associated elastase, MPO (7), α-enolase, or transketolase] makes such a study feasible. While it appears highly likely that the majority of the eDNA is ET-derived, this will pinpoint to an exacerbated innate immune response that fails to target the bacterial colonizer while allowing for the inflammatory milieu that might support the very pathoadaptation of bacteria that represents a partial cause of the etiology of CF symptoms.

A direct consequence of the absence of the CFTR channel is the alteration of the ion balance in extracellular secretions. Calcium metabolism disorder is central to this pathology. CF patients present with significantly elevated concentrations of Ca^2+^ levels in pulmonary fluid and nasal secretions (30, 31). Given the dependence of NETosis on Ca^2+^, it is tempting to speculate that neutrophils in the CF lung environment may tend to NETose more readily compared to neutrophils in the non-CF environment.

**Figure 1:**
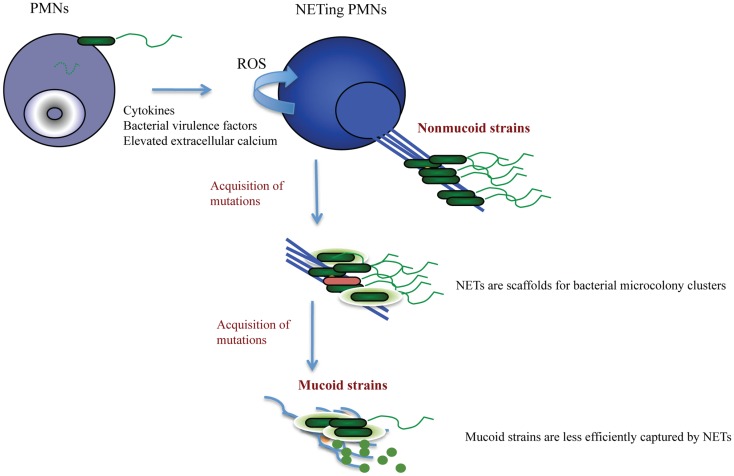
**Proposed scenario for NET-triggered *P. aeruginosa* pathoadaptation**. NETosis is stimulated by *P. aeruginosa*-derived virulence factors released by the non-mucoid “early” strains. The generation of NETs requires activation of NADPH oxidase and ROS production. NETs (blue) provide a scaffold for bacterial attachment and microcolony formation. Albeit sequestering bacteria, NETs fail to successfully kill *Pseudomonas sp*. Only a few dead bacteria (red) are present within the NET-attached microcolonies. The sublytic concentrations of the anti-microbial NET-associated proteins trigger *P. aeruginosa* adaptation and stimulate acquisition of mutations that are associated with polysaccharide production and antibiotic resistance. Consequently, *P. aeruginosa* acquires mucoid phenotype (green bacteria embedded in light green polysaccharide). The mucoid *P. aeruginosa* strains trigger less NETosis due to decreased expression of virulence factors, and may escape from NET trapping by elevated outer membrane vesicle production and DNA shedding.

Cystic fibrosis patients are predominantly colonized with *P. aeruginosa* species. It is thought that this occurs due to the impaired mucocilliary transport that allows for the establishment of chronic bacterial infection in the absence of mechanical clearance. What this scenario does not account for is why there is species-specific dominance. The answer to this question, albeit difficult, was previously sought (32), but multiple scenarios that are not mutually exclusive are possible. A scenario that currently intrigues us is that pathogen sequestration by NET structures promotes bacterial microcolonization, and, as mechanical clearance of these microcolonies fails in CF, the NETosing neutrophils drive bacterial pathoadaptation, thereby having the innate immunity govern colonization. The *Pseudomonas* dominance can be explained by its incredible pathoadaptability to withstand the bactericidal activities of NETs. Data from our group suggest that the non-mucoid *P. aeruginosa* were more efficiently trapped by NETs than the mucoid strains (5, 33). In addition, the non-mucoid strains induced NET formation better than the mucoid strains. Several *Pseudomonas-*derived factors were implicated including flagella and pyocyanin (5, 34). In the case of CF, such NET release may actually support initial bacterial sequestration and drive mutability. Indeed, NETs are equipped with proteins that were implicated in *P. aeruginosa* mutagenesis. A recent insightful research report shows that LL-37, a component of NETs, triggers *P. aeruginosa* mutagenesis (35). Sub-inhibitory concentrations of LL-37 induced mutations within the *mucA* gene to promote antibiotic resistance. Together, these findings highlight potential deficiencies in the NET model of defense, particularly in the context of chronic CF infection, and demonstrate the need to therapeutically address the presence of NETs in CF (Figure 1).

One popular symptomatic treatment option for CF is the administration of recombinant human DNase I (rhDNAse/pulmozyme/dornase alfa) (36) in order to clear the abnormally high levels of eDNA present (11). Clinical evidence shows that treatment with Dornase alfa over a limited period of time ranging from 1 up to 2 years is associated with moderate improvement of lung function. The majority of the Dornase alfa trials include patients whose age range from young children to adults. This makes it difficult to asses whether there is a specific window for treatment where the administration of rhDNase I is most beneficial to patients. If NETs provide a scaffold for bacterial retention, and, hence, promote bacterial colonization, then early treatment with rhDNAse may be more beneficial than late.

Given our current understanding of NET biology, concern exists with regard to the use of DNase I. This is especially raised due to the propensity of DNase I to release NET-resident elastase, which subsequently degrades histones (11). Since histones are NET components that contribute to bacterial adhesion (and therefore sequestration), a decrease in histones may yield a decrease in bacterial sequestration. This is especially significant if bacteria were not effectively killed by NETs in CF, as, in that case, DNase I therapy may, in part, be contributing to the release of pathogenic bacteria and the concomitant exacerbation of infection. One way to address this complication would be to administer an elastase inhibitor along with DNase I; another way would be to reduce the NETosis process altogether by inhibiting an upstream component. There are approaches and candidate drugs to decrease NETosis. For example, chloroquine, an anti-malarial drug, inhibits *in vitro* generation of NETs. Alternatively, PAD4 inhibitors similarly decrease NETosis. Another potential approach to controlling NET release is to engage the Siglec 9 neutrophil surface receptor. Siglecs are a family of immunoglobulin-like cell receptors, of which many have inhibitory cytosolic motif components (ITIMs). Siglec 9 is one such receptor known to have this ITIM element. Once the extracellular domain of Siglec 9 binds with an α2–3 sialic acid linkage of a sialoglycoprotein near the neutrophil cell, the ITIM element signals tyrosine phosphorylation and upregulation of SHP-1 and SHP-2, which leads to secretion of anti-inflammatory cytokines, including IL-10 (37). Perhaps a small molecule, such as a sialated microcarrier or antibody, may allow for Siglec 9-mediated inhibition of NETosis in a more controlled manner. Neither of these options has been currently tested in the CF animal models; however, they may hold promise.

While, initially, NETs are casted to carry a conserved core of associated proteins, what gets deposited on the NET structures defines the outcomes of NET-mediated inflammatory regulation. In SLE, the excessive deposition of IgG onto NETs reduces accessibility of the DNase 1 and, consequently, the timely removal of NETs is impaired, contributing to attendant autoimmune responses. Do NET reservoirs induce autoantibodies in CF? Would the coating of NET fragments in CF with antibodies and complement impair their clearance similarly to SLE? No studies have been undertaken to define the levels of anti-DNA or anti-histone antibody titers in CF so far. However, our initial data suggests that complement C3b is highly deposited on CF-patient derived NET structures, supporting this possible scenario.

In gout, aggregated NET structures carry serine protease enzymatic activities that degrade the proinflammatory cytokines deposited on the NETs, while sparing the NET-associated IL-8 and IL-1RA and, thereby, promote resolution of inflammation (38). This exciting discovery illustrated that, depending on the concentration and composition, NETs stimulate quite a variety of inflammatory responses. Shauer and colleagues suggested that impairment in aggNET formation leads to chronic inflammation. It is tempting to question whether CF-specific NET composition exists to alter the inflammatory milieu and cause chronic inflammation and impaired bacterial clearance. Unfortunately, reports on CF-NET composition during disease exacerbations are limited.

## Concluding Remarks

Although NETs carry a formidable collection of weaponry to sequester and impair the spread of bacteria, they fare better at trapping than killing. NET-mediated immunity relies on mechanical clearance from the mucosal surfaces, and, when this fails, in cases such as CF, NETs may drive bacterial microcolonization. In this sense, the use of DNase I, once bacteria have colonized the lungs, will not be as efficient as when administered much earlier to prevent colonization. Alternatively, novel approaches to regulate NETosis should be discovered to limit excessive eDNA presence and the ensuing inflammatory responses.

## Conflict of Interest Statement

The authors declare that the research was conducted in the absence of any commercial or financial relationships that could be construed as a potential conflict of interest
